# Case Report: A rare case of ocular giant cell arteritis with bilateral orbital inflammation and diagnostic challenges

**DOI:** 10.3389/fneur.2026.1840150

**Published:** 2026-06-09

**Authors:** Alexandru N. Lerint, Oreoluwa E. Morakinyo, Jae Won Oh, Vijaya Lakshmi Valaparla, Xiangping Li

**Affiliations:** Department of Neurology, University of Texas Medical Branch, University of Texas Medical Branch at Galveston, Galveston, TX, United States

**Keywords:** chronic steroid use, ocular giant cell arteritis, orbital inflammation, orbital MRI, vision loss in the elderly

## Abstract

**Background:**

Giant cell arteritis (GCA) is a systemic vasculitis of medium- and large-sized arteries, most commonly presenting with headache, scalp tenderness, and, if untreated, can lead to visual loss from ischemic optic neuropathy. Although the most common ophthalmic manifestations of Giant Cell Arteritis are related to ocular ischemia, orbital involvement can occur and remains underrecognized. Fewer than 10 cases in the literature describe perioptic or retrobulbar infiltration, underscoring the uniqueness and diagnostic challenge of such presentations.

**Case description:**

75-year-old male with past medical history of hypertension, benign prostatic hyperplasia status post transurethral resection, traumatic subdural hematoma (SDH), who presented with episodic left eye vision loss (central and peripheral vision), followed by acute altitudinal vision loss in the right eye for 1 week. He also complained of right-sided headaches s/p SDH and chronic joint pain, but denied any other constitutional symptoms. The neurological exam revealed significantly decreased right eye visual acuity with an altitudinal field defect, color desaturation, and grade 2 + disc edema. Labs revealed an erythrocyte sedimentation rate (ESR) of 21 mm/h (normal age-adjusted), and a C-reactive protein (CRP) of 1.9 mg/dL(elevated). Magnetic resonance imaging of the brain and orbits, with and without contrast, showed bilateral retrobulbar/perioptic soft-tissue infiltration and enhancement, suggestive of an inflammatory, infectious, or autoimmune process. Blood work showed normal thyroid function and negative rheumatological work-up. Spinal fluid studies were within normal limits except for mildly elevated protein (white-blood cell 3, red-blood cell 1, Protein 52, and Glucose 54, while serum glucose was 94). Temporal artery biopsy was positive for temporal arteritis. His hospital course was complicated by left eye episodic vision loss. High-dose IV steroids led to initial improvement and stabilization of symptoms at discharge, requiring oral steroid taper. 10 months later, he had persistent vision loss in the right eye at the follow-up visit and also suffered complications from chronic steroid use.

**Conclusion:**

This case highlights the challenges in diagnosing and treating GCA. Diagnostic delay can occur from under-recognition of atypical presentations. Neuroimaging provided important diagnostic value in this case; however, its routine role in diagnostic criteria requires validation in larger studies.

## Introduction

Giant Cell Arteritis is a granulomatous large-vessel vasculitis that predominantly affects individuals over the age of 50. It occurs more frequently in women and demonstrates marked geographic variation, with higher incidence reported in Northern European populations (1).

Clinically, GCA most often involves branches of the external carotid artery, particularly the temporal artery, and presents with new-onset headaches, scalp tenderness, jaw claudication, and systemic features. Ocular involvement represents a critical complication of GCA, precipitated by inflammatory occlusion of the posterior ciliary arteries. This compromise of the optic nerve vascular supply most frequently manifests as arteritic anterior ischemic optic neuropathy (A- AION), a condition characterized by sudden, often permanent visual impairment without prompt treatment (2).

Although the most common ophthalmic manifestations of Giant Cell Arteritis are related to ocular ischemia, orbital involvement can occur and remains underrecognized. In a recent multicenter case–control study, Guggenberger et al. (3) noted 32% of the GCA patients had abnormal orbital imaging findings (18/56), with optic nerve sheath enhancement being the most frequent abnormality, reported in 13/18 patients (72%). Ophthalmic artery vessel wall enhancement occurred unilaterally in eight patients and bilaterally in three; in contrast, optic nerve parenchymal enhancement was rare, observed in only one patient (4). While bilateral orbital inflammation is a hallmark of our patient’s presentation, this manifestation remains exceedingly rare in the literature, documented almost exclusively in isolated case studies (4).

The 2022 ACR/EULAR classification criteria updated the 1990 version by integrating advanced imaging modalities, including color Doppler ultrasound, axillary involvement, and FDG-PET activity, alongside traditional clinical and laboratory values (5). Despite these updates, significant diagnostic gaps persist: the criteria do not yet incorporate the role of vessel wall MRI, which remains a critical tool for detecting mural edema and thickening in both cranial and extracranial vessels. Furthermore, current protocols still rely heavily on Erythrocyte Sedimentation Rate (ESR) and C-reactive protein (CRP). However, these markers have limitations; in approximately 10–20% of biopsy-proven cases (the confirmatory standard), inflammatory markers remain within normal limits at presentation, representing a critical diagnostic blind spot for clinicians, where current criteria may fail to capture the full spectrum of the disease (6).

In these cases, high-resolution Brain MRI (Magnetic Resonance Imaging) can demonstrate segmental enlargement and enhancement of the temporal arteries, indicating vessel wall inflammation, and serve as a valuable adjunct to clinical and laboratory assessment in confirming the diagnosis (7).

This report contributes to the limited literature on GCA presenting as orbital inflammatory disease, highlighting a unique case characterized by MRI evidence of bilateral retrobulbar infiltration and optic nerve sheath enhancement despite borderline normal inflammatory markers, and highlights orbital MRI clues that may facilitate earlier diagnosis. Moreover, given the significant morbidity associated with prolonged corticosteroid use, this case reinforces the importance of integrating emerging steroid-sparing agents, such as Tocilizumab and JAK inhibitors, into standard care.

## Case description

A 75-year-old man with a history of hypertension, traumatic 3 mm subdural hematoma (SDH, 5 months before presentation, managed conservatively), bilateral LASIK, and cataract extraction with intraocular lens implantation (8 years prior in the right eye; 14 years prior in the left eye), recent BPH s/p transurethral resection of the prostate (TURP), presented with subacute, progressive, painless vision loss. He complained of transient vision loss for 15 to 20 min in the left eye (OS, totally gray, central and peripheral vision) for 1 week, with 1–3 occurrences per day, followed by acute painless altitudinal vision loss in the right eye (OD) for 1 day. He also endorsed residual right temporal headaches radiating to the occiput since the SDH and chronic joint pain. The patient denied diplopia, jaw claudication, temporal tenderness, fevers, night sweats, weight loss, or other focal neurological deficits.

On neuro-ophthalmologic examination, visual acuity was limited to counting fingers (CF) at one foot OD and 20/100 OS, the latter of which improved to 20/20 with pinhole testing. Pupillary assessment revealed symmetric and reactive pupils, notably complicated by a right-sided relative afferent pupillary defect (RAPD). Confrontation and formal visual field testing demonstrated a superior altitudinal defect OD, and 60% color desaturation compared with the left. Fundus examination revealed hyperemic optic disc edema OD, while the optic disc was normal OS. Both eyes showed macular drusen, with otherwise normal retinal vessels and no hemorrhages.

The slit-lamp examination was unremarkable, and intraocular pressures were within the normal range. Ancillary testing, including formal visual field assessment and optical coherence tomography, was not available at the time of presentation. The remainder of the neurological exam was otherwise unremarkable. Additionally, there was no temporal tenderness, cord-like induration, or diminished pulses on temporal artery examination.

Laboratory evaluation revealed a mild elevation of systemic inflammatory markers. The erythrocyte sedimentation rate (ESR) was 21 mm/h (Reference [Ref]: 0–15 mm/h), lower than the age-adjusted upper limit of 37.5 mm/h as calculated by the Miller formula (age/2). The C- reactive protein (CRP) was elevated at 1.9 mg/dL (Ref: <1.0 mg/dL). Platelet level was normal at 264 (Ref 150–328 10*3/μL). Cerebrospinal fluid (CSF) analysis demonstrated an elevated protein level of 52.0 mg/dL (Ref: 15–45 mg/dL) in the presence of a normal leukocyte count (3/μL; Ref: 0–5/μL). The CSF glucose level remained within the normal physiological range at 54 mg/dL, consistent with the expected two-thirds ratio relative to a serum glucose of 94 mg/dL. Extensive serological and CSF screening for infectious and autoimmune etiologies, including aquaporin-4 (AQP4) and myelin oligodendrocyte glycoprotein (MOG) antibodies, was unremarkable.

The ophthalmology and rheumatology services were consulted. At the time of presentation, arteritic anterior ischemic optic neuropathy (AAION) secondary to giant cell arteritis (GCA) was considered; however, the initial clinical suspicion was low given the lack of temporal tenderness or typical constitutional symptoms, normal ESR, and platelet count. Based on the 2022 ACR/EULAR classification clinical criteria, the patient initially yielded a score of 6 (3 points for sudden visual loss and 3 points for elevated CRP); however, given the presence of an alternative explanation for CRP elevation (BPH s/p further TUR), further neuroimaging was pursued.

Magnetic resonance imaging (MRI) of the brain and orbits demonstrated a striking, symmetric infiltration of the bilateral retrobulbar regions, characterized by abnormal enhancement of the optic nerves and their respective sheath complexes; notably, this enhancement extended beyond the dural surface into the adjacent orbital fat (Figure 1). Computed tomography angiography (CTA) confirmed the patency of the intracranial vascular structures, with no evidence of large- vessel vasculitis or aneurysms.

**Figure 1:**
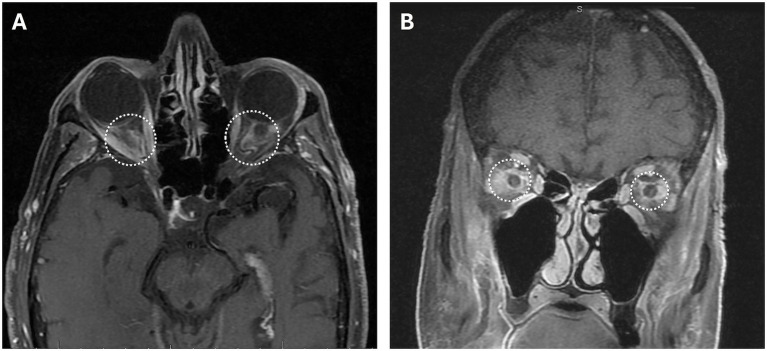
Brain and orbital MRI demonstrates bilateral retrobulbar infiltration and optic nerve sheath enhancement (dashed circles). **(A)** Axial T1-weighted MRI after gadolinium contrast administration. **(B)** Coronal T1-weighted MRI after gadolinium contrast administration.

This constellation of findings represented a significant diagnostic challenge. The extensive retrobulbar infiltration necessitated excluding a broader differential diagnosis. This included infectious optic neuritis, infiltrative neoplastic processes, and idiopathic orbital inflammatory syndrome (IOIS). Furthermore, the severity of the visual loss and the “perioptic” enhancement pattern required the systematic exclusion of sarcoidosis, IgG4-related disease, and demyelinating conditions, specifically neuromyelitis optica spectrum disorder (NMOSD) and MOG-antibody-associated disease (MOGAD), all of which were ruled out in this patient.

Additional MRI findings, including mural enlargement and enhancement of the bilateral superficial temporal arteries, pointed towards Giant Cell Arteritis, and the patient received on the 2nd day of admission an extended course of intravenous methylprednisolone (IVMP) at a dosage of 500 mg twice daily for 5 days and underwent a temporal artery biopsy (TAB) (Figure 2).

**Figure 2:**
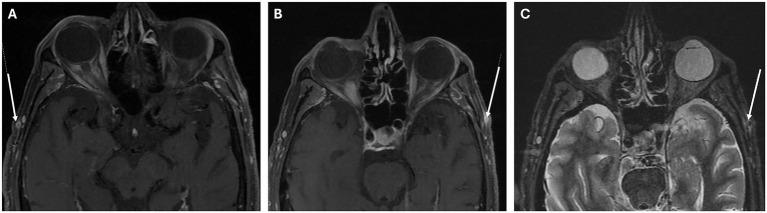
Brain MRI demonstrates temporal (giant cell) arteritis. **(A,B)** T1-weighted fat-saturated post-contrast MRI demonstrates thickening and enhancement of the bilateral superficial temporal artery walls. **(C)** Short tau inversion recovery (STIR) sequence shows perivascular edema (arrow).

Histopathological evaluation of the specimen revealed extensive, patchy inflammatory infiltrates composed of histiocytes and lymphocytes within the muscularis media and intima. Notably, the inflammation involved the internal elastic lamina (IEL), a hallmark finding for temporal arteritis (Figure 3).

**Figure 3:**
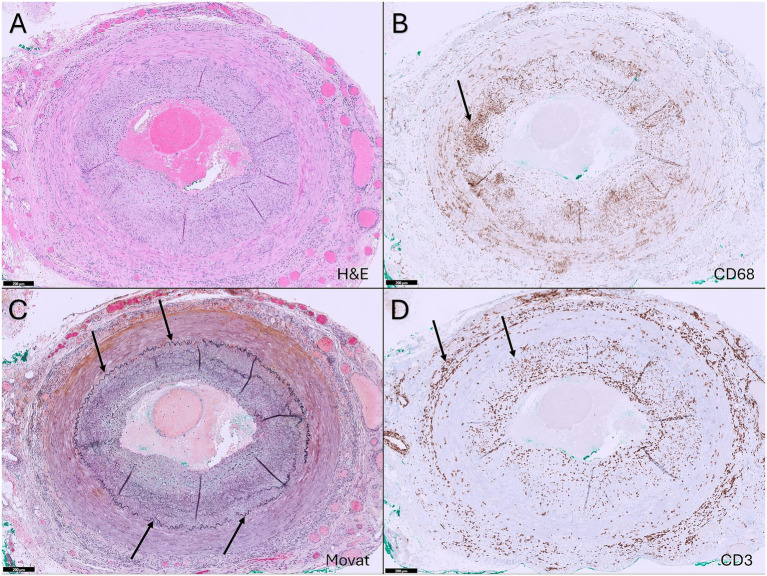
Histologic features of temporal (giant cell) arteritis. **(A)** Sections of the temporal artery revealed T-cell and histiocyte-rich inflammatory infiltrates involving all three layers of the muscular artery (Hematoxylin and eosin, H&E). **(B)** CD68 immunohistochemistry highlights numerous macrophages within the artery wall (arrow), with a predominance in the intima and media. **(C)** Movat pentachrome stain shows damage and fragmentation of the internal elastic lamina (arrows; black staining indicates elastic tissue). **(D)** CD3 immunohistochemistry demonstrates T-cell predominant lymphocytic infiltration of the artery wall (arrows). All images at 50x magnification, scale bars = 200 microns.

During the inpatient stay, the patient experienced episodic left eye vision loss described as “blackout” episodes, which eventually stabilized following the intravenous methylprednisolone (IVMP) course. The patient was discharged on prednisone 80 mg daily with a slow tapering regimen and scheduled for outpatient follow-up with the neuro-ophthalmology and rheumatology services.

The patient was subsequently lost to follow-up and continued corticosteroid therapy under the care of a private optometrist. At the 10-month neuro-ophthalmology evaluation, visual acuity remained stable, though significant deficits persisted. Clinical examination revealed his Best- Corrected Visual Acuity (BCVA) was hand motion (HM) in the right eye (OD) and 20/20 in the left eye (OS). Color vision was 0/14 OD and 1/14 OS. Pupils were regular, symmetrical, and reactive to light with a marked right-sided RAPD. The slit lamp examination showed unremarkable anterior segment findings. Optic disc exam showed marked optic disc pallor with large cup OD and mild pallor OS without active edema. Humphrey visual field test showed generalized depression OD (MD: −27.00) and the nonspecific scattered defects OS (MD: −2.81).

The Optical coherence tomography (OCT) exam showed severe thinning and atrophy OD (GT: 34) and marked thinning nasally and superiorly OS (GT: 51). These OCT findings in the right eye were consistent with optic neuropathy; while not pathognomonic, they most likely represent chronic sequelae of arteritic anterior ischemic optic neuropathy in the setting of biopsy-proven GCA. Additionally, in the interim, the left eye showed optic nerve thinning nasally. At this time of the visit, the patient was on prednisone 10 mg per day. He reported a vertebral fracture and marked skin ecchymosis due to steroid side effects. At 11 months after the discharge, he established care with the rheumatology clinic and was started on Tocilizumab weekly injections in addition to prednisone 5 mg daily.

## Discussion

Giant cell arteritis (GCA) represents the most common systemic vasculitis in individuals over 50 years of age, with potentially devastating ophthalmologic consequences if diagnosis and treatment are delayed. Atypical presentations continue to challenge clinicians and may lead to diagnostic delays with irreversible visual sequelae.

### The limitations of serological markers

The 2022 ACR/EULAR classification criteria for Giant Cell Arteritis (GCA) assign substantial diagnostic weight to systemic inflammatory markers, assigning three points for a C-reactive.

protein (CRP) ≥ 10 mg/L or an erythrocyte sedimentation rate (ESR) ≥ 50 mm/h. While CRP is recognized as a more sensitive predictor of biopsy positivity than ESR, both remain surrogate indicators of a systemic inflammatory response rather than direct evidence of vascular wall pathology. Crucially, literature suggests that 10–15% of biopsy-proven GCA cases present with normal inflammatory markers, creating a significant diagnostic dilemma in patients, such as the one in our case, whose borderline or normal serology may mask the underlying vasculitis (5, 8). Consequently, the absence of an elevated ESR or CRP should not preclude a comprehensive GCA work-up, as diagnostic inertia in these atypical presentations can delay critical corticosteroid intervention and lead to irreversible ischemic complications. Advanced imaging or invasive tests like temporal artery biopsy are often necessary due to a significant lack of specific serological markers in the diagnosis of GCA, although these may not be readily available, particularly in a rural area.

### Atypical neuroimaging and retrobulbar infiltration

This case presents a particularly unusual manifestation of biopsy-proven GCA, characterized by bilateral retrobulbar inflammatory infiltration with perioptic and retrobulbar enhancement seen in the *brain and orbital MRI*. Our patient presented with acute bilateral vision loss with unilateral disc edema, in the absence of classic systemic symptoms, further complicated by imaging findings that mimicked infectious, infiltrative, or demyelinating processes.

While the clinical presence of an altitudinal visual field defect and a relative afferent pupillary defect (RAPD) localized the pathology to the right optic nerve, the MRI findings demonstrated bilateral retrobulbar soft tissue infiltration and enhancement extending well beyond the optic nerve sheath complex. This pattern differs from classic arteritic anterior ischemic optic neuropathy (AAION), which typically exhibits minimal orbital enhancement, and the more circumscribed optic perineuritis commonly reported in GCA.

Recent literature supports the high prevalence of these objective imaging findings. Eldaya et al. (9) demonstrated that orbital MRI abnormalities occur in 69% of biopsy-proven GCA patients, frequently presenting bilaterally, and are not restricted to the symptomatic orbit. Our case corroborates an evolving paradigm suggesting that GCA-related neuro-ophthalmic disease extends beyond traditional ischemia to include primary periorbital and perioptic inflammation (OPN). This presentation expands the clinical spectrum by demonstrating broad retrobulbar infiltration transcending the nerve sheath or extraocular muscles, a pattern that mirrors documented phenotypes of extensive posterior involvement.

### The role of neuroimaging

In cases where inflammatory markers remain equivocal, MRI plays a pivotal role in identifying temporal artery enlargement and enhancement. Neuroimagingallows for the localization of segments with intense mural inflammatory changes, which may serve to determine the optimal site for biopsy and potentially reduce the incidence of false-negative specimens (7).

Furthermore, a recent systematic review and meta-analysis by El Yaman et al. (10) suggested that MRI demonstrates a superior and more consistent diagnostic yield compared to Color Doppler ultrasound (CDUS). While CDUS remains efficacious in specialized, high-volume centers, its utility is significantly circumscribed by inter-observer variability and inherent operator dependence. The objective, cross-sectional nature of MRI offers a more reproducible diagnostic standard, particularly in diverse clinical settings where specialized sonographic expertise may be unavailable. It would be reasonable to suggest that the MRI evidence should be incorporated into the future diagnostic criteria for Giant Cell Arteritis (GCA).

### Limitations of treatment in real-life settings

High-dose glucocorticoids remain the mainstay of GCA treatment, yet they carry significant associated toxicity and do not restore lost function. A major limitation illustrated by this case is the irreversibility of ischemic visual injury despite appropriate corticosteroid therapy. Although the patient received high-dose intravenous methylprednisolone (500 mg twice daily for 5 days) followed by an oral prednisone taper, his best-corrected visual acuity (BCVA) remained at hand motion at 10 months, with severe retinal nerve fiber layer thinning on OCT, color vision of 0/14 in the right eye, and profound visual field depression. This is consistent with prior literature demonstrating that once arteritic anterior ischemic optic neuropathy (AAION) is established, corticosteroids primarily aim to prevent further ischemic injury or contralateral involvement rather than restore vision (9).

While the delayed transition to tocilizumab was largely precipitated by a 10-month loss to follow-up, this outcome nevertheless highlights the broader challenges of managing GCA in real-world settings. The patient was distressed about the significant morbidity observed in his case, including vertebral fracture and marked skin ecchymosis, which illustrates the consequences of prolonged corticosteroid exposure in the context of a logistical barrier. This underscores the importance of timely biologic initiation, given that tocilizumab has been shown in randomized trials to reduce both relapse rates and cumulative glucocorticoid exposure.

## Conclusion

This case highlights an atypical presentation of biopsy-proven giant cell arteritis (GCA) manifesting as extensive bilateral retrobulbar and perioptic inflammation despite borderline inflammatory markers. Such a phenotype expands the recognized clinical spectrum of GCA- associated orbital disease and underscores the necessity of maintaining a high index of suspicion in elderly patients presenting with apparent idiopathic orbital inflammation.

In the absence of classic systemic symptoms or significant serological markers, neuroimaging can serve as a critical diagnostic tool, providing objective evidence of mural enlargement and enhancement of the temporal arteries. Furthermore, MRI can facilitate targeted localization for temporal artery biopsy, potentially reducing the incidence of false-negative results. Ultimately, this case emphasizes unmet needs in both diagnosis and treatment of GCA.

## Data Availability

The original contributions presented in the study are included in the article/supplementary material, further inquiries can be directed to the corresponding author.
